# A Comprehensive Review of the Endometrial Receptivity Array in Embryo Transfer: Advancements, Applications, and Clinical Outcomes

**DOI:** 10.7759/cureus.67866

**Published:** 2024-08-26

**Authors:** Swati M Dahiphale, Deepika Dewani, Jayashree M Dahiphale, Manjusha Agrawal, Apoorva Dave, Sandhya Pajai, Garapati Jyotsna

**Affiliations:** 1 Obstetrics and Gynaecology, Jawaharlal Nehru Medical College, Datta Meghe Institute of Higher Education & Research, Wardha, IND; 2 Neurology, Fortis Hospital, Mumbai, IND

**Keywords:** personalized medicine in ivf, recurrent implantation failure (rif), implantation window, assisted reproductive technologies (art), embryo transfer, endometrial receptivity array (era)

## Abstract

Embryo transfer is a pivotal procedure in assisted reproductive technologies (ART). Yet, the success of this process hinges on multiple factors, with endometrial receptivity playing a critical role in determining the likelihood of successful implantation. The endometrial receptivity array (ERA) is an advanced diagnostic tool designed to personalize embryo transfer timing by assessing the endometrium's receptivity. This review comprehensively examines the ERA, exploring its biological foundation, technological development, and clinical applications. The ERA's ability to analyze the expression of genes associated with endometrial receptivity offers a tailored approach to identifying the optimal window of implantation (WOI), particularly benefiting patients with recurrent implantation failure (RIF) or repeated unsuccessful in vitro fertilization (IVF) cycles. Clinical outcomes from ERA-guided embryo transfers indicate improvements in implantation rates and overall pregnancy success, although challenges such as result variability and cost-effectiveness persist. This review also discusses the latest advancements in ERA technology, including integrating genomic and transcriptomic analyses, non-invasive techniques, and using artificial intelligence (AI). Controversies regarding the widespread application of ERA and its necessity in all IVF cases are critically examined. By summarizing the current state of ERA in embryo transfer, this review aims to inform clinicians, researchers, and patients about its potential to enhance ART outcomes and to highlight areas for future research and innovation.

## Introduction and background

Embryo transfer is a crucial component of assisted reproductive technologies (ART), serving as the final and decisive step in the series of procedures to achieve a successful pregnancy [1]. This process involves carefully transferring a fertilized embryo into the uterus, which ideally implants and progresses into a healthy pregnancy. Embryo transfer is most commonly associated with in vitro fertilization (IVF), wherein eggs are retrieved from a woman’s ovaries, fertilized with sperm in a laboratory setting, and subsequently cultured into embryos [2]. Once these embryos reach an appropriate stage of development, they are transferred into the woman’s uterus during the optimal period for implantation [2]. Over the years, embryo transfer has seen significant advancements, with improvements in embryo culture media, selection techniques, and transfer protocols leading to higher success rates. Despite these advancements, implantation remains one of the most challenging aspects of ART, as many variables influence the likelihood of success. Factors such as embryo quality, uterine environment, and endometrial receptivity are all crucial in determining whether the embryo will successfully implant. Thus, understanding and optimizing these factors are essential for enhancing ART outcomes and increasing the chances of a successful pregnancy [3].

Endometrial receptivity is a fundamental factor in the successful implantation of an embryo and, consequently, in achieving a successful pregnancy. This concept refers to the period during which the endometrium, the lining of the uterus, is optimally prepared to receive and support the implantation of an embryo. This period, often referred to as the window of implantation (WOI), typically occurs between days 19 and 23 of a 28-day menstrual cycle [4]. During this narrow receptive window, the endometrium undergoes complex molecular, cellular, and structural changes that create a favorable environment for embryo implantation. These changes are driven by a delicate interplay of hormones, cytokines, and growth factors, which work together to make the endometrium receptive to the implanting embryo. Any disruption in this process can result in implantation failure, a leading cause of infertility and recurrent pregnancy loss [5]. Therefore, accurately assessing endometrial receptivity is critical for the success of ART. Traditional methods for evaluating endometrial receptivity, such as histological dating of endometrial biopsies, have precision and predictive value limitations. As a result, more advanced tools, such as the endometrial receptivity array (ERA), have been developed to provide a more reliable and personalized approach to determining the optimal timing for embryo transfer [6].

The ERA is a pioneering diagnostic tool that offers a highly precise evaluation of endometrial receptivity. Developed as a molecular diagnostic test, the ERA analyzes the expression of a specific set of genes associated with endometrial receptivity, providing a personalized assessment of the WOI for each patient [6]. The ERA test involves taking an endometrium biopsy during a specific phase of the menstrual cycle, which is then analyzed using advanced genomic technologies. The gene expression profile obtained from the biopsy is compared against a reference profile of receptive and non-receptive endometrium, allowing for the determination of whether the endometrium is in a receptive state or if adjustments are needed in the timing of embryo transfer [7]. Since its introduction, the ERA has become increasingly utilized in clinical practice, particularly for patients with a history of recurrent implantation failure (RIF) or those who have experienced multiple unsuccessful IVF cycles. By personalizing the timing of embryo transfer to align with the patient’s specific implantation window, the ERA can potentially improve implantation rates and overall pregnancy outcomes in ART. This personalized approach marks a significant advancement in the field, offering hope to many patients who have struggled with infertility [8].

The primary objective of this review is to provide a comprehensive overview of the endometrial receptor array (ERA) within the context of embryo transfer. This review aims to explore the biological foundations of endometrial receptivity, the development and technical aspects of the ERA, and its applications in clinical practice. Additionally, the review will examine the clinical outcomes associated with ERA-guided embryo transfer, highlighting this approach's potential benefits and limitations. Furthermore, the review will discuss the latest advancements and innovations in ERA technology, address the challenges and controversies surrounding its use, and explore potential future directions in this rapidly evolving field. Through this review, we seek to provide clinicians, researchers, and patients with a deeper understanding of the role of the ERA in optimizing embryo transfer and improving ART outcomes.

## Review

Endometrial receptivity: biological background

Definition and Phases of Endometrial Receptivity

Endometrial receptivity refers to the critical period during the menstrual cycle when the endometrium, the inner lining of the uterus, is optimally prepared for the implantation of an embryo. This receptivity is crucial for achieving a successful pregnancy, as it determines the ability of a fertilized egg to attach and embed itself within the uterine lining [4]. The implantation process is not instantaneous; it progresses through meticulously coordinated phases. Initially, the blastocyst, which is the early-stage embryo, hatches from its protective shell and approaches the endometrium. Next, the blastocyst undergoes apposition, aligning closely with the endometrial surface, followed by adhesion, which involves forming molecular interactions that firmly anchor the blastocyst to the endometrium. Finally, penetration and invasion occur, allowing the embryo to embed itself within the endometrial tissue, establishing a connection that will eventually lead to the development of the placenta. This complex sequence highlights the importance of precise timing and the specific physiological changes required within the endometrium to enable successful implantation [9].

Molecular and Genetic Markers of Receptivity

The molecular and genetic landscape of the endometrium plays a crucial role in determining its receptivity. Various markers reflect the intricate hormonal interplay that prepares the endometrium for implantation. Among these, the expression of estrogen receptor-alpha (ER-α) and progesterone receptor (PR) is particularly significant, as these receptors are key in mediating the effects of ovarian hormones. The presence of ER-α and PR ensures that the endometrium responds appropriately to hormonal signals, promoting receptivity [10]. Factors such as COUP-TFII, a transcription factor, also suppress pathways that might inhibit implantation. At the same time, BCL6, a gene associated with immune regulation, is overexpressed during the receptive phase. These molecular markers collaborate to create an environment conducive to embryo implantation, underscoring the complex biological processes that support endometrial receptivity [11].

The Window of Implantation

The WOI is a crucial period during which the endometrium is optimally receptive to an implanting blastocyst [12]. In humans, this window generally occurs between days 19 and 21 of the menstrual cycle, corresponding with the luteal phase when progesterone levels peak. However, the WOI can vary between individuals, with some women experiencing shifts in this window due to factors such as hormonal imbalances or underlying reproductive disorders [13]. In ART, including IVF, the precise timing of embryo transfer is critical. Transferring an embryo outside the WOI can significantly decrease the likelihood of successful implantation. Understanding the specifics of the WOI enables clinicians to customize embryo transfer protocols for individual patients, thereby improving the chances of achieving a successful pregnancy [1].

Factors Affecting Endometrial Receptivity

**Figure 1:**
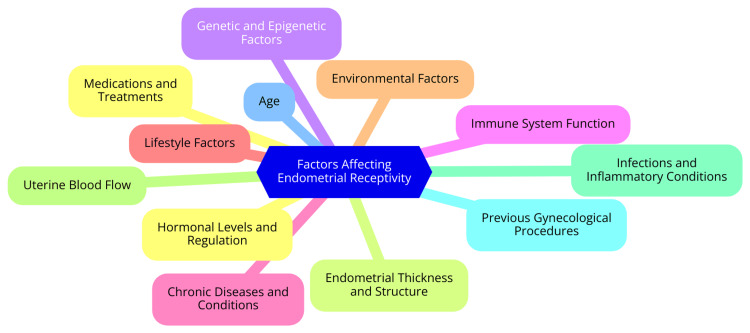
Factors affecting endometrial receptivity Image Credit: Dr SM Dahiphale

Numerous factors can influence endometrial receptivity, affecting the likelihood of successful implantation. Hormonal regulation is critical, with estrogen and progesterone playing essential roles in preparing the endometrium for implantation. An optimal balance of these hormones is necessary for maintaining endometrial health and receptivity. Additionally, the physical characteristics of the endometrium, such as its thickness, significantly impact receptivity [14]. A well-developed endometrial lining is typically associated with higher implantation rates, whereas a thin endometrium may present challenges. Endometrial pathologies, such as endometriosis or polyps, can disrupt normal receptivity and lead to implantation failure. Other factors, including the timing of embryo transfer relative to the WOI, maternal age, and overall reproductive health, are also crucial in determining endometrial receptivity. By understanding these variables, healthcare providers can better evaluate and optimize successful implantation conditions, ultimately enhancing fertility treatment patients' outcomes [15]. Factors affecting endometrial receptivity are shown in Figure 1.

The development of the ERA

Historical Background and Initial Research

The ERA was developed to address the need for more effective strategies for IVF, particularly for women experiencing RIF. Historically, our understanding of the WOI has advanced considerably. Early research underscored the importance of the endometrium in successful embryo implantation, but traditional methods for assessing receptivity, such as histological evaluations, often produced inconsistent and subjective results [16]. These limitations led researchers to investigate molecular techniques that could better evaluate endometrial receptivity more precisely. The introduction of the ERA was a significant advancement in reproductive medicine, utilizing gene expression profiling to pinpoint the optimal timing for embryo transfer. This approach has improved the likelihood of successful implantation and enhanced pregnancy outcomes for IVF patients [17].

Technical Aspects of ERA: How It Works

The ERA utilizes advanced microarray technology to assess the expression of approximately 248 genes associated with endometrial receptivity [18]. The procedure starts with a biopsy of the endometrial lining, which is performed during the presumed WOI, typically around days 19-21 of a natural cycle or after five to six days of progesterone administration in an artificial cycle [19]. After obtaining the biopsy, RNA is extracted and analyzed using microarray technology to quantify gene expression levels. The results classify the endometrium into one of three states: receptive, pre-receptive, or post-receptive. This classification provides clinicians with precise guidance on the optimal timing for embryo transfer, thereby significantly enhancing the likelihood of successful implantation. The molecular approach of the ERA offers greater accuracy and reproducibility compared to traditional histological assessments, making it a valuable tool for personalized embryo transfer (pET) strategies [20].

Comparison With Traditional Methods of Assessing Endometrial Receptivity

Traditional methods for assessing endometrial receptivity have largely depended on histological evaluation, which examines morphological changes in the endometrium during the WOI. These methods have been criticized for their subjectivity and variability, often resulting in inconsistent interpretations and outcomes. In contrast, the ERA offers an objective and quantitative assessment of gene expression, enabling a more precise identification of the WOI [21]. Studies have shown that the ERA’s accuracy surpasses traditional histological methods, significantly enhancing diagnostic capabilities for endometrial receptivity. Using a molecular approach, the ERA deepens the understanding of endometrial biology and improves clinical decision-making, ultimately leading to higher success rates in IVF procedures [6].

Evolution and Refinement of the ERA Technique

Since its introduction, the ERA technique has experienced significant evolution and refinement. Initially, the focus was establishing gene expression profiles associated with endometrial receptivity. Recent advancements, however, have aimed at enhancing the assay’s sensitivity and specificity. Ongoing research explores the impact of various factors, such as hormonal treatments, patient age, and individual endometrial characteristics, on receptivity [7]. These refinements are essential for optimizing the ERA's clinical application, ensuring it accurately guides embryo transfer timing and improves reproductive outcomes for IVF patients. As the technology progresses, the ERA is expected to become increasingly pivotal in personalized reproductive medicine, offering new hope to those facing difficulties in achieving successful pregnancies [22].

Applications of ERA in clinical practice

The ERA has notable applications in clinical practice, particularly for optimizing embryo transfer timing, addressing RIF, and enhancing overall IVF success rates [18]. The ERA enables precise identification of the WOI by analyzing the expression of 238 genes associated with endometrial receptivity. This allows for pET, where the timing of embryo transfer is tailored to each patient's specific endometrial profile. Research shows that pET guided by ERA results can improve pregnancy rates by ensuring embryos are transferred when the endometrium is most receptive, thereby maximizing the chances of successful implantation [22]. For patients experiencing RIF, the ERA proves particularly beneficial. Studies indicate that a significant proportion of these patients have a displaced WOI, which can lead to unsuccessful embryo transfers. Using the ERA to identify the WOI accurately, clinicians can adjust the timing of transfers, leading to improved implantation and ongoing pregnancy rates. Research has shown that patients with a receptive ERA have outcomes comparable to those who undergo pET after a non-receptive ERA, underscoring the importance of personalized timing in overcoming implantation challenges [22].

The ERA is valuable for optimizing embryo transfer timing even in patients with normal uterine anatomy. The WOI can still vary, and utilizing the ERA ensures that transfers are conducted during the optimal window, enhancing the likelihood of successful implantation and pregnancy outcomes. For patients with endometrial pathologies, such as a thin endometrium or other uterine abnormalities, the ERA is particularly crucial. It helps determine if the endometrium is receptive, guiding the timing of embryo transfers. Studies have shown that even with a thin endometrium, a significant percentage of patients may have a receptive endometrium, allowing for successful pET and improved pregnancy rates [18]. Incorporating the ERA into IVF protocols has been associated with improved success rates. By personalizing the embryo transfer process, the ERA aligns the timing of the transfer with the physiological readiness of the endometrium. Meta-analyses have demonstrated that ERA results significantly enhance pregnancy rates compared to conventional frozen embryo transfer methods, especially in populations with a history of implantation failure. This personalized approach not only increases the chances of pregnancy but also contributes to better overall clinical outcomes [18].

Clinical outcomes of ERA-guided embryo transfer

Impact on Pregnancy Rates

One of the most notable advantages of ERA-guided embryo transfer is its substantial impact on pregnancy rates, particularly for women experiencing RIF. Research indicates pET based on ERA results can significantly enhance clinical pregnancy rates [8]. In a multicenter randomized controlled trial, ERA-guided transfers achieved a pregnancy rate of 72.5%, compared to 54.3% in the control group that did not use ERA guidance. Other studies have supported these findings, with clinical pregnancy rates reaching up to 51.7% for patients undergoing ERA-guided transfers [23]. However, it is important to recognize that outcomes can vary. Some studies have reported no significant differences in pregnancy rates between ERA-guided transfers and standard procedures in certain populations. This variability highlights the necessity of individualized treatment approaches tailored to each patient's unique characteristics [23].

Effect on Live Birth Rates

The impact of the ERA on live birth rates is a crucial aspect of its clinical utility. A comprehensive Cochrane review found that ERA-guided transfers are associated with increased live birth rates, rising from 33.3% to 40.2% [24]. Additionally, recent research has shown that cumulative live birth rates after 12 months were significantly higher in the ERA group than those who did not undergo ERA testing, with rates of 71.2% versus 55.4% [23]. These findings suggest that the ERA can significantly enhance the chances of achieving live births from successful pregnancies. However, some retrospective analyses have indicated that ERA-guided pET may not consistently improve live birth rates compared to fresh or frozen embryo transfers without ERA guidance. This variability underscores the need for further research to establish more definitive conclusions about the ERA's role in improving live birth outcomes [18].

Reducing the Incidence of Miscarriage

The potential of the ERA to reduce miscarriage rates is an area of ongoing research. Although some studies suggest that ERA-guided transfers could improve outcomes, the evidence of its impact on miscarriage rates remains inconclusive [25]. For example, one study reported miscarriage rates of 15.2% in the ERA group compared to 13.2% in the non-ERA group, with no statistically significant difference observed [26]. This indicates that while the ERA may help in optimizing the timing of embryo transfers, its effect on miscarriage rates is not yet definitively established. Given that miscarriage is a multifaceted issue influenced by various factors, further research is needed to clarify the relationship between ERA-guided transfers and miscarriage incidence [26].

Patient Satisfaction and Psychological Impact

The psychological and emotional dimensions of undergoing fertility treatments are significant and cannot be overlooked. Patient satisfaction and psychological outcomes associated with ERA-guided embryo transfer are vital components of the overall treatment experience [27]. The personalized approach of the ERA can enhance patient confidence in the transfer process, potentially leading to increased satisfaction levels. However, the psychological impact can vary widely among individuals, especially for those grappling with the stress and disappointment of RIFs. While specific data on patient satisfaction related to ERA is somewhat limited, the general trend suggests that personalized treatments often improve the overall patient experience. This approach can foster a sense of empowerment and hope, contributing positively to the emotional well-being of patients [22].

Case Studies and Real-World Evidence

Real-world evidence and case studies provide valuable insights into the effectiveness of ERA-guided transfers. For instance, a retrospective review conducted in Canada found higher ongoing pregnancy rates among patients who underwent ERA-guided transfers compared to those who did not [28]. Additionally, individual case studies have documented successful pregnancies following ERA-guided transfers in complex cases, demonstrating the practical utility of this method in clinical settings. However, it is important to acknowledge reports of less favorable outcomes in certain populations, suggesting that while ERA may enhance results for many, it is not universally effective. This variability highlights the need for further research to understand better the overall effectiveness of ERA across different clinical scenarios [29].

Advancements and innovations in ERA technology

Integrating the ERA with genomic and transcriptomic technologies has significantly advanced its diagnostic capabilities. By combining ERA with next-generation sequencing (NGS), researchers can analyze a broader spectrum of genetic markers and expression profiles. This enhanced approach offers a more comprehensive understanding of the molecular mechanisms driving endometrial receptivity and could lead to the discovery of new biomarkers for predicting implantation success [30]. Recent innovations in non-invasive techniques have further improved ERA testing by reducing the need for invasive endometrial biopsies. Methods such as analyzing menstrual fluid or urine samples for gene expression have been explored, aiming to provide similar diagnostic accuracy while minimizing patient discomfort and procedural risks [31].

Artificial intelligence (AI) and machine learning (ML) are increasingly used to analyze ERA data. These technologies can uncover patterns and correlations within complex datasets, potentially leading to more accurate predictions of endometrial receptivity. By training algorithms on extensive datasets, AI can assist clinicians in optimizing the timing of embryo transfers and enhancing clinical outcomes [32]. Future research in ERA technology will likely focus on several key areas. Efforts will be directed toward refining the ERA to offer even more personalized treatment plans based on individual genetic and environmental factors. Additionally, researchers are exploring the application of ERA in contexts beyond IVF, such as managing endometrial disorders and fertility preservation strategies. Longitudinal studies will be essential to validate the long-term benefits of ERA-guided embryo transfers and assess its impact on overall reproductive health. Future advancements may also involve integrating ERA with other fertility treatments, such as preimplantation genetic testing (PGT), to improve embryo selection [33]. These developments hold promise for enhancing reproductive outcomes and personalizing fertility treatments, ultimately contributing to higher success rates in ART [1]. Table 1 summarizes the advancements and innovations in ERA technology.

**Table 1:** Advancements and innovations in ERA technology AI, artificial Intelligence; ML, machine learning; ERA, endometrial receptivity array

Advancement/innovation	Description	Impact/benefits
Integration with genomic and transcriptomic technologies	Utilization of advanced genomic and transcriptomic tools to refine the ERA’s gene expression profiles.	Enhances the accuracy and precision of endometrial receptivity assessments, leading to more personalized treatment plans.
Development of non-invasive techniques	Research into non-invasive methods such as uterine aspirates or fluid samples to assess endometrial receptivity.	Reduces the need for invasive biopsies, improving patient comfort and accessibility of the ERA test.
Artificial intelligence and ML models	Application of AI and ML to analyze ERA data and predict receptivity patterns more effectively.	Improves predictive accuracy and the ability to personalize embryo transfer timing based on complex data analysis.
Advanced bioinformatics tools	Use of sophisticated bioinformatics algorithms to interpret complex ERA data and integrate it with other patient-specific factors.	Enhances data interpretation and integration, leading to more refined and individualized treatment recommendations.
Integration with other ART technologies	Combining ERA with other ART advancements, such as embryo monitoring technologies and advanced culture systems.	Provides a more comprehensive approach to ART, optimizing both embryo quality assessment and endometrial receptivity.
Development of ERA for diverse patient populations	Research into adapting ERA for use in various populations, including those with endometrial pathologies or atypical cycles.	Expands the applicability of ERA to a broader range of patients, potentially improving outcomes in diverse clinical scenarios.

Challenges and controversies

The ERA has attracted considerable attention for its potential to improve embryo transfer success rates in IVF. However, its implementation and effectiveness face several challenges and controversies [18]. One major challenge with the ERA is the variability in test results and their interpretation. The ERA classifies endometrial receptivity into receptive, pre-receptive, or post-receptive states based on gene expression profiles. However, studies have shown that results can vary significantly between cycles for the same patient, raising concerns about the test's reliability. Intra-patient variability complicates decision-making, as a non-receptive result in one cycle may not be consistent in subsequent cycles, especially with changes in hormonal treatments. This inconsistency raises questions about the ERA's clinical utility in guiding embryo transfer timing [34]. The cost-effectiveness of the ERA is also debated. The test typically costs between $850 and $1,000, a significant expense compared to the overall cost of an IVF cycle. Critics argue that while the ERA may benefit some patients, particularly those with RIF, its routine use in all IVF cases may not be justified due to insufficient evidence demonstrating improved live birth rates in broader patient populations. The financial burden could also deter patients from accessing potentially beneficial treatments [35].

The personalization of embryo transfer through the ERA raises ethical questions. While the aim is to enhance reproductive success, tailoring treatment based on genetic profiling can lead to dilemmas regarding the definition of "normal" endometrial receptivity. Some concerns about genetic markers might overshadow other crucial factors influencing implantation success, such as overall reproductive health and psychological well-being. Moreover, the commercialization of such tests raises ethical issues about informed consent and the potential exploitation of vulnerable patients seeking solutions to infertility [36]. Finally, the necessity of the ERA in all IVF cases remains a contentious issue. Proponents argue that the test can significantly improve outcomes for patients with a history of implantation failure. However, many experts believe that most IVF patients do not require such testing, as standard protocols often produce satisfactory results without the added complexity and cost of the ERA. Some studies suggest that routine use of the ERA does not improve live birth rates in the general IVF population, indicating that its application should be selective rather than universal. This debate underscores the need for further research to determine which patient groups might benefit most from the ERA [7]. Table 2 summarizes the challenges and controversies associated with ERA technology.

**Table 2:** Challenges and controversies associated with ERA technology. RIF, recurrent implantation failure; ERA, endometrial receptivity array

Challenge/controversy	Description	Implications
Variability in ERA results	Inconsistencies in ERA results due to differences in laboratory techniques, sample handling, or data interpretation.	Can lead to variability in clinical decision-making and affect patient outcomes.
Cost-effectiveness	High cost of ERA testing compared to traditional methods of assessing endometrial receptivity.	Raises questions about the economic justification for widespread ERA use, especially in resource-limited settings.
Clinical necessity	Debate over whether ERA is necessary for all patients or only those with RIF.	Uncertainty about the benefits of ERA for routine use versus its targeted application in specific patient populations.
Ethical considerations	Concerns about the ethical implications of using genomic data to influence reproductive decisions.	Raises questions about patient consent, data privacy, and potential pressure to undergo additional interventions.
Interpretation of results	Challenges in interpreting ERA results due to variability in individual endometrial responses and gene expression patterns.	May lead to misinterpretation of data and inappropriate clinical recommendations.
Integration with clinical practice	Difficulties in integrating ERA findings with existing ART protocols and practices.	Challenges in establishing standardized guidelines for incorporating ERA results into clinical decision-making.

## Conclusions

In conclusion, the ERA represents a significant advancement in the field of ART, offering a personalized approach to embryo transfer that has the potential to enhance implantation success and improve overall pregnancy outcomes. By precisely identifying the WOI through the analysis of gene expression profiles, the ERA allows for a more tailored timing of embryo transfer, particularly benefiting patients with RIF or previous unsuccessful IVF cycles. While the ERA has shown promise in clinical practice, it is not without its challenges, including variability in results, cost considerations, and ongoing debates regarding its necessity for all patients undergoing IVF. Nonetheless, as research continues to advance, the ERA is likely to play an increasingly important role in reproductive medicine, offering new possibilities for optimizing fertility treatments. Future developments in ERA technology, coupled with a deeper understanding of endometrial receptivity, will further refine this tool, potentially leading to even better outcomes for patients seeking to overcome infertility.
